# Relationship Between Occupational Noise and Hypertension in Modern Enterprise Workers: A Case–Control Study

**DOI:** 10.3389/ijph.2022.1604997

**Published:** 2022-11-04

**Authors:** Bo Zhou, Yueyan Lan, Yifei Bi, Chaoxiu Li, Xiaohong Zhang, Xiaomei Wu

**Affiliations:** ^1^ Department of Clinical Epidemiology and Center of Evidence Based Medicine, The First Hospital of China Medical University, Shenyang, Liaoning, China; ^2^ College of Foreign Languages, University of Shanghai for Science and Technology, Shanghai, China; ^3^ Department of Clinical Epidemiology, The Fourth Hospital of China Medical University, Shenyang, Liaoning, China

**Keywords:** risk factors, blood pressure, dose response relationship, cumulative noise exposure, noise level

## Abstract

**Objective:** The association between occupational noise exposure and hypertension is controversial. Thus, we aimed to assess the relationship between occupational noise exposure and hypertension.

**Methods:** This was a case‒control study, and 509 cases and 1,018 controls from an automobile company were included between July and October 2013. Occupational noise exposure was defined as exposure to noise level ≥80 dB(A) (Lex, 8 h) or cumulative noise exposure (CNE) ≥ 80 dB(A)-years. To assess the associations of noise level and CNE with hypertension, univariate and multivariate logistic regression were performed to calculate odds ratios (ORs) and 95% confidence intervals (CIs). The restricted cubic spline function was used to establish dose‒response curves.

**Results:** A noise level ≥80 dB (A) (Lex, 8 h) was significantly associated with hypertension (OR 2.48, 95% CI 1.89–3.24). CNE ≥80 dB (A)-years was significantly associated with hypertension (OR 1.53, 95% CI 1.18–2.00). Nonlinear relationships between noise level, CNE and hypertension were found (p- nonlinear<0.05).

**Conclusion:** Our study suggests that occupational noise exposure is a potential risk factor for hypertension in automobile company workers.

## Introduction

Cardiovascular diseases (CVDs) are the leading cause of death in China [1, 2]. Hypertension is not only associated with an increased risk of CVD, but also the most important risk factor for morbidity from all causes worldwide [3]. A Chinese national survey showed that the overall prevalence of hypertension among individuals over 18 years of age was 27.5% in 2020 [4]. Different studies have estimated the prevalence of hypertension as 26.5%–33.18% among workers in China [5, 6]. According to the 2017 National Workforce Survey, 400 million individuals were workers, and the number of workers appears to increase annually [7]. According to the results of the Fourth National Economic Census and the National Survey on the Status of Occupational Disease Hazards, noise exposure is a common occupational risk factor for occupational disease. In China, 32.6 million workers exposed to noise, which may lead to hearing problems in more than 9 million people [8]. China has implemented a series of laws and standards to control occupational noise exposure [9, 10]. However, with the development of industrialization, noise exposure, which can result in adverse health effects, is difficult to avoid.

A meta-analysis reported that the pooled prevalence of any occupational exposure to noise was 17% i 108,256 participants in 38 countries [11]. Noise pollution was found to be a risk factor for and trigger of hypertension [12, 13]. A previous review indicated that noise exposure can initiate physiological stress responses, which lead to a cascade of effects, including increases in heart rate and stress hormone levels (cortisol, adrenalin, and noradrenaline). This may ultimately lead to pathophysiological alterations in the intermediate or long term [14]. An animal model showed that exposure to low levels of aircraft noise for 1–4 days caused an increase in the release of stress hormones and in circulating angiotensin II levels, with a significant stress-induced increase in blood pressure, and increased oxidative stress within the plasma and vasculature caused vascular (endothelial) dysfunction [15]. Human studies showed that exposure to noise increased the levels of plasma noradrenaline and angiotensin II, leading to endothelial dysfunction and stimulation of the renin-angiotensin system, thus increasing blood pressure (BP) [16, 17]. A meta-analysis of 23 studies indicated that noise exposure significantly increased the risk of hypertension by a factor of 1.72 [18]. Previous studies reported that workers exposed to high noise levels had a significantly higher risk of hypertension than those exposed to low noise levels [19–21]. In contrast, Stokholm et al. reported that the risk of hypertension did not increase in individuals with noise exposure levels in the lower half of the 80–90 dB (A) range [22]. Noise exposure was associated with more clinically severe hypertension, but not with uncomplicated hypertension [23].

The association between occupational noise exposure and hypertension is controversial. Therefore, a case–control study was carried out to analyse the association between occupational noise exposure and hypertension. This study provides evidence of the relationship between occupational noise exposure and the risk of hypertension.

## Methods

The company in this study is involved in the manufacturing and assembly of automobiles. According to the International Standard Industrial Classification of All Economic Activities (ISIC) and the International Standard Classification of Occupations (ISCO), workers in our study were categorized into Industrial sector 29 (specify ISIC-4 code) and Occupation 7231, 7224, 7212, 7214 (specify ISCO-08 code) groups. All participants worked on the production line. The job duties of the production line included painting, drive shaft machining and maintenance, welding of motor bodies and accessories, grinding and polishing, emissions testing, etc. Noise was mainly generated from motor paint removal, supersonic wave destruction tests, drive shaft machining and the use of electric wrenches and cutting machines. The types of noise included steady-state noise and impulse noise.

**FIGURE 1:**
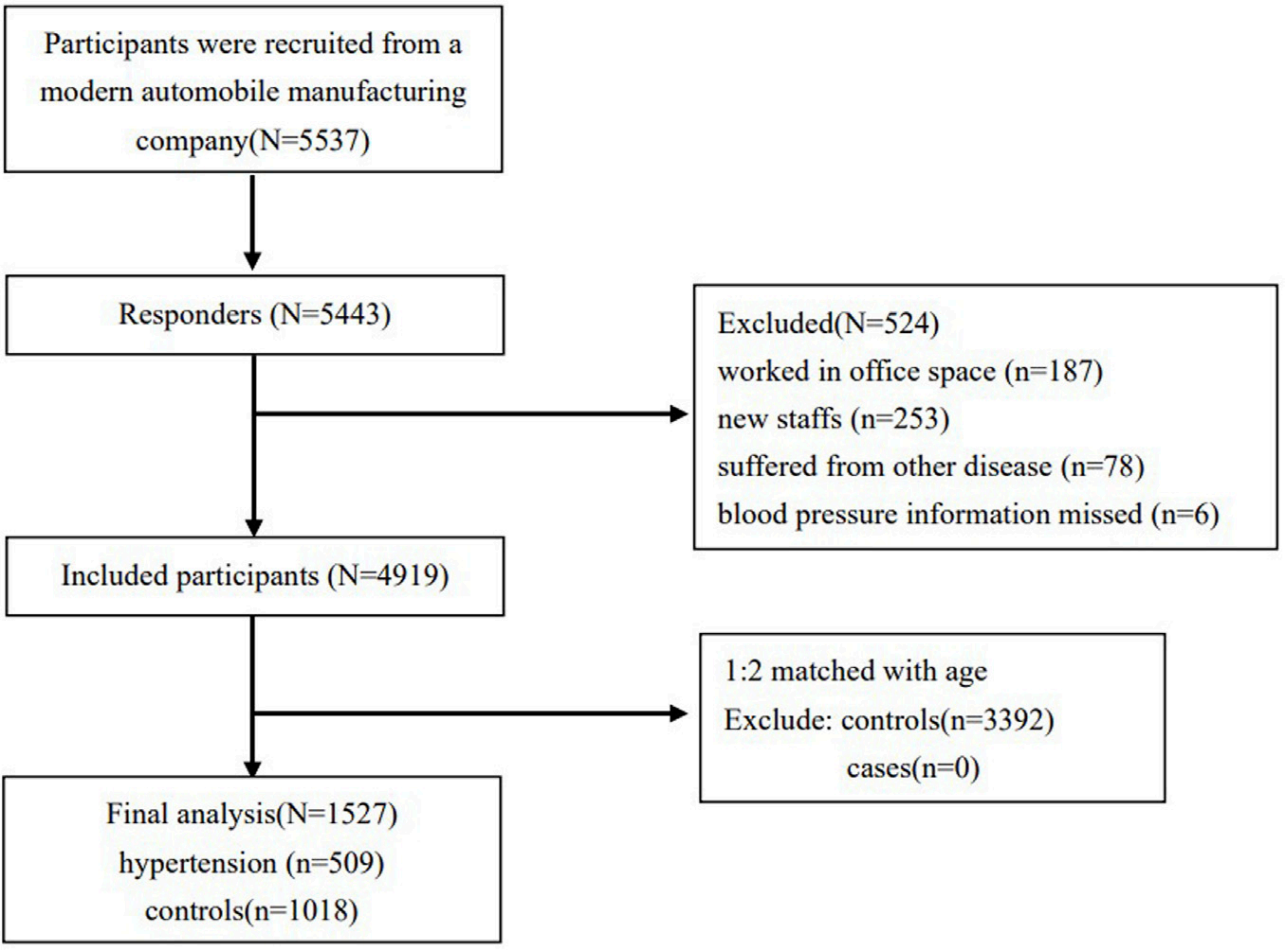
Study design and flow diagram (China, 2013).

This was a case‒control study. A total of 5,537 participants were recruited from a modern automobile manufacturing company in Shenyang, Liaoning Province. Among the 5,537 participants, 5,443 individuals completed occupational physical examinations between July and October 2013, and the response rate was 98%. We excluded participants who worked in an office space where with no occupational noise, for example, in the purchasing department, sales department, and property management department (N = 187). New staff who participated in the physical examination were excluded because these individuals may not have been exposed to occupational noise yet (N = 253). People suffering from other cardiovascular and cerebrovascular diseases, chronic tumours, liver and kidney diseases, etc. (N = 78), and those missing blood pressure information (N = 6) were excluded. After these exclusions, 4,919 participants were included in the analysis. All subjects included in this study were male. Participants who met the diagnostic criteria were included in the case group. Participants who were not diagnosed with hypertension according to the same criteria were enrolled in the control group. Case‒control matching by age was performed at a ratio of 1:2 among 509 hypertension cases and 4,410 controls. Thus, the final analytical sample included 1,527 participants (Figure 1). Comparisons of the included and excluded groups showed that smoking status, family history of hypertension, duration of noise exposure, noise level and cumulative noise exposure (CNE) were not significantly different between the two groups; body mass index (BMI), total cholesterol (TC) levels, triglyceride (TG) levels, high-density lipoprotein (HDL) levels, low-density lipoprotein (LDL) levels, and heart rate were significantly different between the two groups (Supplementary File).

### Assessment and Definition of Hypertension

Hypertension was measured and defined according to the guidelines for the prevention and treatment of adult hypertension (2010) in China [24]. All participants were required to fast overnight before blood pressure measurement. Participants rested in a chair with back support for approximately 10 min, before their blood pressure was measured with their arms supported at heart level. Measurements were repeated 1–2 min apart, and the average was recorded. If the difference between the two readings of systolic blood pressure (SBP) or diastolic blood pressure (DBP) readings was more than 5 mmHg, measurement was repeated, and the average of the three readings was recorded. Measurements were performed by trained physicians with a regularly calibrated electronic automated device (HEM-746C, Omron, Japan).

SBP ≥140 mmHg and/or DBP ≥90 mmHg without the use of antihypertensive drugs was diagnosed as hypertension (*n* = 494); patients with a history of hypertension, and those currently taking antihypertensive medication, were diagnosed as having hypertension even though their blood pressure measurement was below 140/90 mmHg (*n* = 15).

### Assessment and Definition of Noise Exposure

Occupational noise exposure was evaluated with an audio device (AWA6218; Beijing Xihua Instrument Technology Co., Ltd.) by qualified industrial hygienists from measurement companies. The evaluation of noise exposure was carried out using equivalent continuous dB (A) weighted sound pressure levels (Lex, 8 h), as recommended by the Occupational Health Standard of the People’s Republic of China: Measurement of Noise in the Workplace [GBZ/T 189.8–2007] guidelines (China, 2007). The measurement locations were established according to the sound distribution in the workshop. The measurement instrument was fixed on a tripod, and the microphone was placed at the height of a worker’s ear (1.50 m for standing, 1.10 m for sitting). The measurements were repeated 3 times and were performed 10 min for steady-state noise and 20 min for impulse noise, and the average was recorded. CNE was calculated as CNE = 10 × log (10^SPL/10^ × years of noise exposure), where SPL is the sound pressure level [dB (A)] of occupational noise exposure [22]. Occupational noise exposure was defined as exposure to a noise level ≥80 dB (A) (Lex, 8 h) or CNE ≥80 dB (A)-years.

According to Factors of Hazardous Substances in Industrial Sites--Physical Factors [GBZ2.2-2007] (China, 2007), steady-state noise was defined as a noise level fluctuation of no more than 3 dB (A) during the measurement period. Impulse noise was defined as a sudden noise burst and ended quickly, with a duration <0.5 s, an interval time >1 s and a change >40 dB (A).

All the devices passed the quality standards of the metrology department, and were verified by the Metrology Department of Liaoning Province, China.

### Covariates

We considered the following covariates in this analysis: age, smoking status, family history of hypertension, biochemical indices, dust exposure, toxin exposure, the duration of noise exposure, and BMI. BMI was calculated as weight (in kilograms) divided by the square of the height (in metres). The categorical definitions of underweight (BMI<18.5), normal weight (BMI of 18.5–23.9), and overweight (BMI≥24.0) followed the recommendations of the Working Group on Obesity in China for the Chinese population [25]. Blood sample collection and blood tests were performed by professionals with automatic biochemical instruments. Blood samples collected on site were stored in a refrigerator at − 20°C for biochemical analysis, and with anticoagulant in a refrigerator at − 80°C for DNA extraction.

Measurements of dust and toxin exposure were conducted according to the Occupational Health Standard of the People’s Republic of China: specifications of air sampling for hazardous substance monitoring in the workplace [GBZ 159–2004] guidelines (China, 2004). Dust exposure was evaluated with a dust sampler (DS-21B, DFC-3BT; Shanghai Biaozhuo Instrument Technology Co., Ltd.), and weighed by electronic scales (AG285; Mettler Toledo). Toxin exposure was evaluated with an atmospheric sampler (QT-2A; Beijing Tianyue Instrument Technology Co., Ltd.). The time-weighted average (TWA) was used in the measurements. The TWA was calculated considering an occupational exposure time of 8 h per day.

### Statistical Analysis

SPSS version 24.0 (IBM Corp., Armonk, New York, United States) and R version 4.2.1 (R Foundation for Statistical Computing, Vienna, Austria) were used for statistical analyses. The Kolmogorov‒Smirnov test was used to check whether the continuous variables conformed to a normal distribution. Continuous variables are expressed as means and interquartile ranges. Categorical variables are expressed as numbers of cases and percentages. For the comparison of continuous baseline characteristics, Mann‒Whitney U tests were performed. For categorical variables, the χ2 test was used for comparison. The continuous variables were converted to categorical variables to ensure the existence of a clinically relevant balance by selecting the corresponding cut-off value.

Confounding variables were identified by univariate logistic regression. Odds ratios (ORs) and 95% confidence intervals (CIs) were calculated to investigate the relationships of noise levels and CNE with hypertension by multivariable logistic regression. In this study, three analysis models were developed considering the baseline covariates: 1) a crude model without adjustment; 2) the main model, which was adjusted for family history of hypertension, smoking, BMI, toxin exposure and dust exposure; and 3) the fully adjusted model, which was further adjusted for TC levels, TG levels, HDL levels and LDL levels (the main model and fully adjusted model of noise level were additionally adjusted for duration of noise exposure). Moreover, we assessed the relationship of noise levels and CNE with hypertension by distribution quartile, with the 1st quartile as the reference group. Sensitivity analysis was performed to investigate the association of a noise level ≥85 dB (A) (Lex, 8 h) and CNE ≥85 dB(A)-years with hypertension. Furthermore, sensitivity analysis was repeated after excluding participants who were diagnosed with a history of hypertension or were currently taking antihypertensive medication. To explore the dose‒effect relationships of occupational noise exposure and CNE with hypertension, the dose‒response relationship was evaluated by using a restricted cubic spline (RCS) function model with 3 knots. A two-sided *p* < 0.05 was considered statistically significant.

## Results

The basic characteristics of the 1,527 subjects (case group, 509 individuals; control group, 1,018 individuals) are shown in Table 1. Participants in the study were all male and had a mean age of 29 [25, 32] years. Toxin exposure, CNE, noise level, BMI, TC level, TG level, LDL level and heart rate were significantly different between the two groups (*p* < 0.05). The other factors, including age, duration of noise exposure, smoking, family history of hypertension, dust exposure, HDL level and noise type were not significantly different.

**TABLE 1:** Baseline characteristics of study participants (China, 2013).

	Total N = 1,527	Hypertension N = 509	Normotension N = 1,018	*p*
Age (years)	29 (25, 32)	29 (25, 32)	29 (25, 32)	0.975
Duration of noise exposure (years)	2 (1, 4)	2 (1, 6)	2 (1, 4)	0.096
CNE dB(A)-years	81.81 (78.80, 85.87)	82.97 (78.80, 86.62)	81.81 (78.80, 85.14)	<0.001*
Noise level dB (A) (Lex, 8 h h)	78.80 (75.90, 80.60)	78.80 (78.80, 80.90)	78.80 (75.60, 78.80)	<0.001*
BMI (kg/m^2^)	24.77 (22.15, 27.76)	27.47 (24.69, 29.41)	23, 76 (21.22, 26.12)	<0.001*
TC (mmol/L)	4.6 (4.0, 5.2)	4.9 (4.2, 5.5)	4.4 (3.9, 5.1)	<0.001*
TG (mmol/L)	1.44 (0.91, 2.19)	1.78 (1.20, 2.78)	1.27 (0.86, 1.92)	<0.001*
HDL (mmol/L)	1.15 (0.93, 1.19)	1.02 (0.90, 1.14)	1.07 (0.94, 1.22)	0.045
LDL (mmol/L)	2.58 (2.14, 3.08)	2.78 (2.33, 3.24)	2.48 (2.07, 2.96)	<0.001*
Heart rate (beats/min)	80 (73, 88)	83 (76, 93)	79 (72, 86)	<0.001*
Smoking (yes)	889 (58.2%)	301 (59.1%)	588 (57.8%)	0.608
Family history of hypertension (yes)	1,505 (98.6%)	500 (98.2%)	1,005 (98.7%)	0.448
Dust exposure (yes)	881 (57.7%)	274 (53.8%)	607 (59.6%)	0.031*
Toxin exposure (yes)	923 (60.4%)	377 (74.1%)	546 (53.6%)	<0.001*
Noise type				0.641
impulse	21 (1.4%)	6 (1.2%)	15 (1.5%)	
steady	1,506 (98.6%)	503 (98.8%)	1,003 (98.5)	

Note: Age, duration of noise exposure, CNE, noise level, BMI, TC, TG, HDL and LDL were expressed as median and 25th-75th percentile; smoking, family history of hypertension, dust exposure, toxin exposure, noise type and heart rate were expressed as number of case and percentages.

Abbreviation: CNE: cumulative noise exposure, TG: triglyceride, HDL: high-density lipoprotein, LDL: low-density lipoprotein, TC: total cholesterol, BMI: body mass index.

*p < 0.05.

Table 2 shows the ORs and 95% CIs for hypertension associated with each variable in the univariate regression model. Baseline variables that were considered relevant (e.g., smoking and family history of hypertension) or that showed a significant relationship with the outcome in the univariate analysis were identified as potential cofounding factors. Duration of noise exposure, toxin exposure, dust exposure, BMI, TC level, TG level, HDL level and LDL level were entered into the multivariate regression model.

**TABLE 2:** Hypertension and blood pressure-related factors were identified by univariate logistic regression (China, 2013).

Variable	N	Case group (N, %)	Control group (N, %)	OR (95%CI)	*p*
Noise type					
impulse noise (Ref)	21	6 (1.2)	15 (1.5)	1	0.642
steady-state noise	1,506	503 (98.8)	1,003 (98.5)	1.25 (0.48–3.25)	
Duration of noise exposure					
≤4 years (Ref)	1,151	377 (74.1)	774 (76.0)	1	0.018*
>4 years	376	132 (25.9)	244 (24.0)	1.31 (1.05–1.64)	
Toxin exposure					
no (Ref)	604	132 (25.9)	472 (46.4)	1	<0.001*
Yes	923	377 (74.1)	546 (53.6)	2.47 (1.96–3.12)	
Dust exposure					
no (Ref)	646	235 (46.2)	411 (40.4)	1	0.031*
yes	881	274 (53.8)	607 (59.6)	0.79 (0.64–0.98)	
Smoking					
no (Ref)	638	208 (40.9)	430 (42.2)	1	0.608
yes	889	301 (59.1)	588 (57.8)	1.06 (0.85–1.31)	
Family history of hypertension					
no (Ref)	22	9 (1.8)	13 (1.3)	1	0.450
yes	1,505	500 (98.2)	1,005 (98.7)	0.72 (0.31–1.69)	
BMI (kg/m^2^)					
18.5–23.9(Ref)	46	4 (0.8)	42 (4.1)	1	<0.001*
<18.5	569	86 (16.9)	483 (47.4)	1.87 (0.65–5.35)	0.243
≥24.0	912	419 (82.3)	493 (48.4)	8.92 (3.17–25.0)	<0.001*
TC (mmol/L)					
<5.18(Ref)	1,072	305 (59.9)	767 (75.3)	1	<0.001*
5.18–6.21	362	146 (28.7)	216 (21.2)	1.70 (1.33–2.18)	<0.001*
≥6.22	93	58 (11.4)	35 (3.4)	4.17 (2.68–6.47)	<0.001*
TG (mmol/L)					
<1.70(Ref)	929	244 (47.9)	685 (67.3)	1	<0.001*
1.70–2.25	244	82 (16.1)	162 (15.9)	1.42 (1.05–1.92)	0.023
≥2.26	354	183 (36.0)	171 (16.8)	3.00 (2.33–3.88)	<0.001*
HDL (mmol/L)					
<1.04(Ref)	732	276 (54.2)	456 (44.8)	1	0.001*
1.04–1.54	746	224 (44.0)	522 (51.3)	0.71 (0.57–0.88)	0.002*
≥1.55	49	9 (1.8)	40 (3.9)	0.37 (0.18–0.78)	0.009*
LDL (mmol/L)					
<3.37(Ref)	1,304	402 (79.0)	902 (88.6)	1	<0.001*
3.37–4.13	183	83 (16.3)	100 (9.8)	1.86 (1.36–2.55)	<0.001*
≥4.14	40	24 (4.7)	16 (1.6)	3.37 (1.77–6.41)	<0.001*
Heart rate (beats/min)					
60–99(Ref)	1,378	432 (84.9)	946 (92.9)	1	<0.001*
<60	39	6 (1.2)	33 (3.2)	0.40 (0.17–0.96)	0.04*
≥100	110	71 (13.9)	39 (3.8)	3.99 (2.65–5.99)	<0.001*

Abbreviation: Ref: reference value, OR: odds ratio, CI: confidence interval, CNE: cumulative noise exposure, TG: triglyceride, HDL: high-density lipoprotein, LDL: low-density lipoprotein, TC: total cholesterol, BMI: body mass index.

*p < 0.05.

After adjusting for confounding factors, the ORs and 95% CIs for hypertension associated with noise level and CNE are shown in Table 3. In the crude model without adjustment, a noise level ≥80 dB (A) (Lex, 8 h) was associated with hypertension (OR: 2.54, 95% CI: 2.02–3.19). CNE ≥80 dB (A)-years was also associated with hypertension (OR: 1.56, 95% CI: 1.24–1.97). There were positive associations of noise level and CNE with hypertension in the three models. A noise intensity ≥80 dB (A) (Lex, 8 h) was associated with hypertension (multivariate regression, main model: OR: 2.48, 95% CI: 1.92–3.21). A CNE level≥ 80 dB(A)-years was also associated with hypertension (multivariate regression, main model: OR: 1.51, 95% CI: 1.17–1.95).

**TABLE 3:** Association of noise level, cumulative noise level and hypertension (China, 2013).

Variable	Crude model	Main model^a^	Full adjusted model^b^
	OR (95% CI)	OR (95% CI)	OR (95% CI)
Noise level≥80 dB(A) (Lex,8 h)	2.54 (2.02–3.19)	2.48 (1.92–3.21)	2.48 (1.89–3.24)
Noise level≤75.9 dB(A) (Lex,8 h) (Ref)	1	1	1
75.91–78.8	1.46 (1.09–1.95)	1.41 (1.03–1.92)	1.37 (1.00–1.88)
78.81–80.6	3.29 (2.22–4.87)	2.80 (1.83–4.28)	2.66 (1.73–4.08)
>80.61	3.57 (2.56–4.97)	3.66 (2.52–5.30)	3.45 (2.36–5.03)
CNE ≥80 dB(A)-years	1.56 (1.24–1.97)	1.51 (1.17–1.95)	1.53 (1.18–2.00)
CNE≤78.8 dB(A)-years (Ref)	1	1	1
78.8–81.81	1.48 (1.09–2.02)	1.48 (1.06–2.07)	1.40 (1.00–1.97)
81.82–85.87	3.29 (2.22–4.87)	1.60 (1.15–2.21)	1.55 (1.11–2.15)
85.88-	3.57 (2.56–4.97)	1.46 (1.06–2.01)	1.32 (0.95–1.82)

^a^ Family history of hypertension, smoking, BMI, toxin exposure, dust exposure and duration of noise exposure were adjusted for noise level. Family history of hypertension, smoking, BMI, toxin exposure and dust exposure were adjusted for CNE.

^b^ Family history of hypertension, smoking, toxin exposure, dust exposure, duration of noise exposure, TC, TG, HDL and LDL were adjusted for noise level. Family history of hypertension, smoking, toxin exposure, dust exposure, TC, TG, HDL and LDL were adjusted for CNE.

Abbreviation: TG: triglyceride, BMI: body mass index, TC: total cholesterol, HDL: high-density lipoprotein, LDL: low-density lipoprotein, CNE: cumulative noise exposure.

**FIGURE 2:**
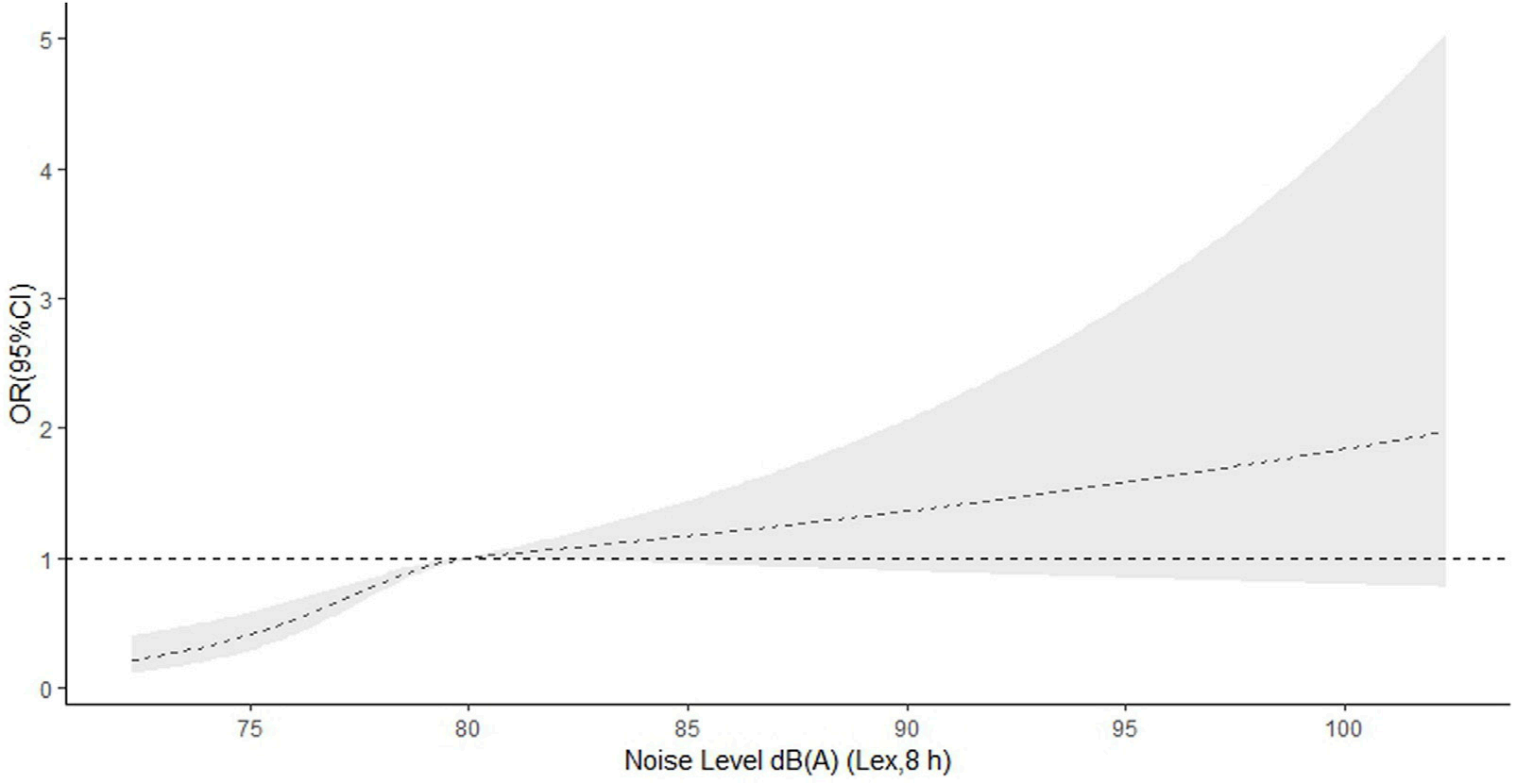
Dose‒response relationship of noise level and hypertension (China, 2013).

**FIGURE 3:**
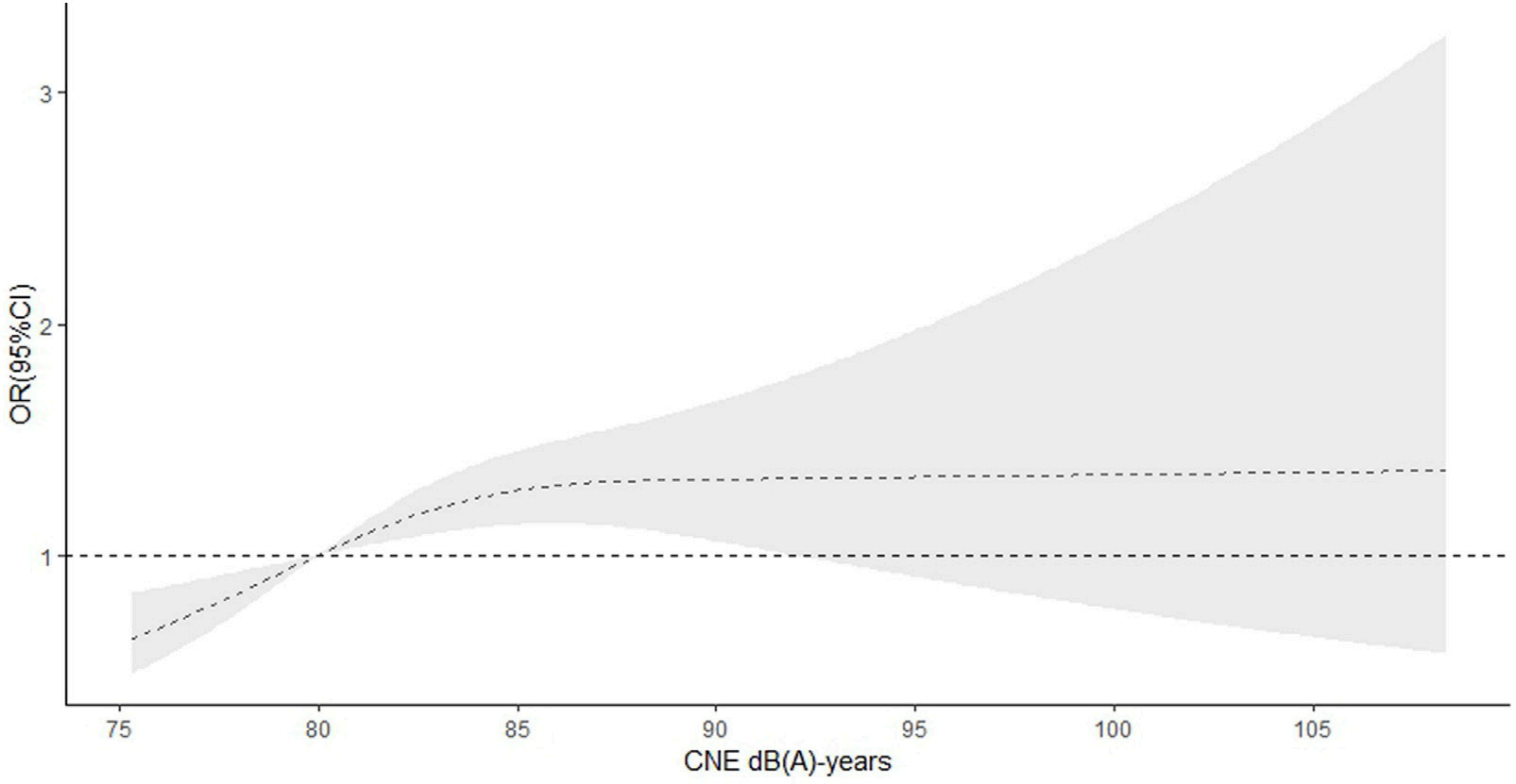
Dose‒response relationship of cumulative noise exposure and hypertension (China, 2013).

In the RCS function model with nonlinear terms, a nonlinear dose‒response relationship was found between occupational noise level and hypertension (p-nonlinear <0.001 in main model). When the noise level was <80 dB(A) (Lex, 8 h), the OR for hypertension increased with increasing noise level and slowly decreased when the noise level was ≥80 dB(A) (Lex, 8 h) (Figure 2). There was also a nonlinear dose‒response relationship between the CNE level and hypertension (p-nonlinear = 0.014 in main model). The OR for hypertension increased until the CNE level reached approximately 85 dB (A)-years, and then began to decrease, with the OR still greater than 1 (Figure 3).

There were no interactions between noise level and potential confounding factors. However, the associations between CNE and smoking (p-interaction = 0.040) and dust exposure (p-interaction = 0.024) were significant (Supplementary File).

The sensitivity analysis showed that a noise level ≥85 dB(A) (Lex, 8 h) was not significantly associated with hypertension. We found a weak relationship between a CNE level ≥85 dB(A)-years and hypertension after further adjustment for TC, TG, HDL and LDL. A noise level ≥80 dB (A) (Lex, 8 h) and CNE ≥80 dB (A)-years were also associated with hypertension after excluding participants who had a history of hypertension or were currently taking antihypertensive medication (Supplementary File).

## Discussion

Our study showed a positive association between occupational noise level and hypertension, in individuals exposed to an occupational noise level ≥80 dB(A) (Lex, 8 h). Consistent with previous research, a meta-analysis reported that the impact of high-intensity noise exposure on workers’ cardiovascular systems was much higher than that in the unexposed control group in China [26]. Chang et al. found a positive relationship between a noise exposure level ≥85 dB(A) and the incidence of hypertension [27]. Another study found that workers exposed to ≥85 dBA had a 7% higher risk of acquiring hypertension; however, the risk ratio (RR) was nonsignificant [28]. We found no association between occupational noise exposure and the risk of incident hypertension or even an inverse association between occupational noise exposure and hypertension [29, 30]. The controversial conclusion may be due to differences in noise exposure measurement methods, study designs, and adjustments for confounding factors. In addition, we found a nonlinear association between occupational noise level and hypertension (*p* < 0.001). The OR for hypertension increased with an increasing noise level when the noise level was <80 dB(A) Lex, 8 h, and slowly decreased when the noise level was >80 dB(A) (Lex, 8 h). The widespread use of hearing-protection devices at work could lead to an underestimation of the association between occupational noise level and hypertension. However, Chen et al. reported associations between exposure to different noise exposure levels and hypertension, and found no evidence suggesting a nonlinear association between noise and the risk of hypertension, with moderate heterogeneity [31]. Sixteen studies reported a linear association per 10 dB(A) of noise, and a risk of hypertension was not observed, with substantial heterogeneity [32]. However, most of the sixteen studies had a cross-sectional design and a considerable risk of bias. Thus, further prospective cohort studies are needed to confirm the dose‒response relationship between occupational noise level and the risk of hypertension.

We also found a positive, statistically significant association between CNE and hypertension. CNE was a strong predictor of hypertension and the exposure-response trends were significant [33]. In addition, Chen et al. found a significant correlation between CNE and blood pressure [34]. Our study showed a nonlinear association between CNE level and hypertension (*p* < 0.05), the OR for hypertension increased rapidly until a CNE level of approximately 85 dB(A)-years and then decreased gradually. Su et al. also reported a nonlinear dose‒response relationship between the level of CNE and hypertension; the OR for hypertension increased rapidly with increasing CNE levels in a range of 82–94 dB(A)-years and then decreased, with a CNE level of <81 dB (A)-years as the reference [35]. Hearing-protection and the survivor effect may account for the decrease in the OR for hypertension risk.

The models in our study were adjusted for air pollution, which is known to be correlated with noise and thus could confound the association between noise and hypertension. Common chemicals used in the automobile manufacturing industry include benzene, toluene, xylene, nitrogen dioxide (NO_2_), carbon monoxide (CO), sulphur dioxide, etc. Cai et al. reported that short-term exposure to NO_2_, ozone (O_3_), and CO as well as long-term exposure to nitrogen oxides (NO_x_) and sulphur monoxide (SO) were positively correlated with hypertension, and may increase the risk of hypertension [36]. In addition, Gan et al. showed that there were independent effects of traffic-related air pollution indicated by the effect of black carbon concentrations on coronary heart disease mortality [37]. Hypertension is a multifactorial polygenic disorder with a tendency to interact with certain environmental factors. Further research is needed to evaluate the confounding and combined effects of air pollution and noise exposure on the risk of hypertension.

Participants in this study were young adults, with a mean age of 29 [25, 32] years. Studies have reported that CVD risk and signs of subclinical CVD are increased in young adults who are prehypertensive [38]. Compared with those with normal blood pressure, young adults with hypertension before the age of 40 years have an increased risk of subsequent CVD events [39, 40]. The hypertension attributable CVD burden in young adults has greatly increased [41]. Thus, strategies are needed to prevent hypertension in young adults who are exposed to noise.

### Strengths and Limitations

We used two noise indicators (CNE and noise level) to estimate the association of occupational noise exposure with hypertension. However, this study has several limitations. All subjects in the study were men, so the conclusions are limited to men. In addition, we were unable to obtain information about previous occupational exposure, traffic-related air pollution and the use of hearing-protection. Therefore, the results could be overestimated. The sample size of participants exposed to noise levels greater than 85 dB (A) (Lex, 8 h) was small. The distribution of noise levels and CNE are shown in histograms (Supplementary File). This weakened the correlation relative to what could be obtained from a large population. Therefore, the results of the sensitivity analysis may be inaccurate.

### Conclusion

Our study suggests that occupational noise exposure is a potential risk factor for hypertension in automobile company workers. There were nonlinear relationships between noise level and CNE and hypertension.

## Data Availability

The datasets generated during and/or analyzed during the current study are available from the corresponding author on reasonable request.
