# Supplementary material for: Relationship Between Occupational Noise and Hypertension in Modern Enterprise Workers: A Case–Control Study
Source: Int J Public Health. 2022 Nov 4;67:1604997. doi: 10.3389/ijph.2022.1604997 (PMC9671941; doi:10.3389/ijph.2022.1604997)
Supplement: Supplementary file 1 [file DataSheet1.docx]

**International Journal of Public Health**

**Relationship between occupational noise and hypertension in modern enterprise workers: a case–control study**

Table 1 The comparation of baseline characteristics between included and excluded groups (China 2013).

|  | Inclusion  N = 1527 | Exclusion  N = 3392 | P |
| --- | --- | --- | --- |
| Age (years) | 29(25,32) | 26(24,30) | <0.001* |
| Duration of noise exposure (years) | 2(1,4) | 2(1,4) | 0.194 |
| CNE dB(A)-years | 81.81(78.80,85.87) | 81.80(78.80,85.10) | 0.989 |
| Noise level dB (A) (Lex,8 h) | 78.80(75.90,80.60) | 78.80(78.20,80.60) | 0.169 |
| BMI (kg/m^2^) | 24.77(22.15,27.76) | 24,22(21.51,26.93) | <0.001* |
| TC (mmol/L) | 4.6(4.0,5.2) | 4.5(3.9,5.1) | <0.001* |
| TG (mmol/L) | 1.44(0.91,2.19) | 1.30(0.87,2.00) | <0.001* |
| HDL (mmol/L) | 1.15(0.93,1.19) | 1.07(0.94,1.21) | <0.001* |
| LDL (mmol/L) | 2.58(2.14,3.08) | 2.52(2.11,2.97) | 0.001* |
| Heart rate (beats/min) | 80(73,88) | 79(72,87) | 0.001* |
| Smoking (yes) | 889(58.2%) | 1958(57.7%) | 0.608 |
| Family history of hypertension (yes) | 1505(98.6%) | 3344(98.6%) | 0.448 |

Note: Age, the duration of noise exposure, CNE, noise level, BMI, TC, TG, HDL and LDL were expressed as median and 25th-75th percentile; smoking, family history of hypertension and heart rate were expressed as number of case and percentages.

Abbreviation: CNE: cumulative noise exposure, TG: triglyceride, HDL: high-density lipoprotein, LDL: low-density lipoprotein, TC: total cholesterol, BMI: body mass index.

* P＜0.05


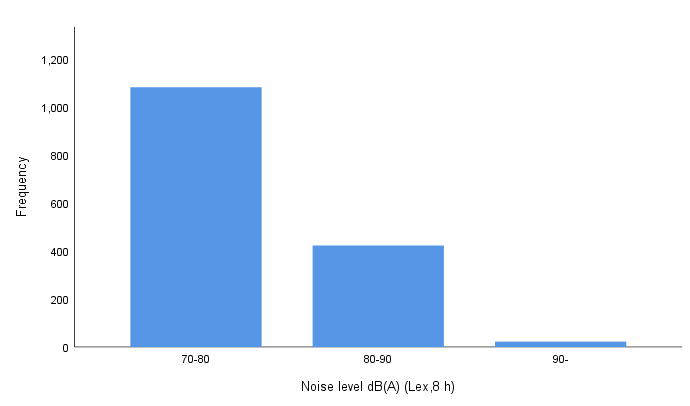


Figure 1 the histogram of noise level dB(A) (Lex,8 h) (China, 2013).


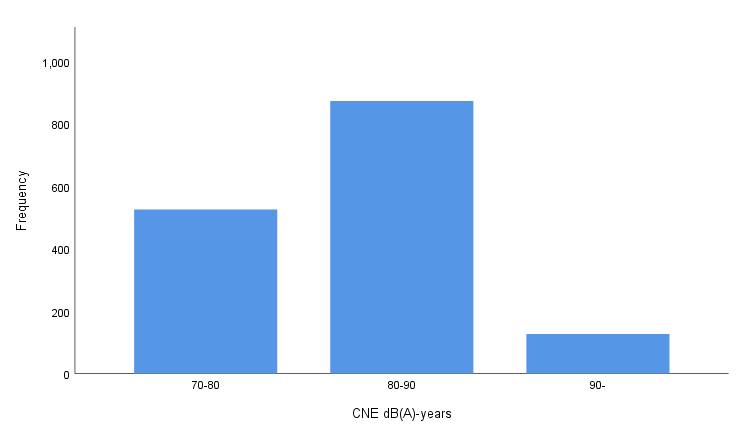


Figure 2 the histogram of cumulative noise exposure dB(A)-year (China, 2013).

Table 2 Sensitivity analysis of noise level and cumulative noise exposure with hypertension (China, 2013).

|  | Crude model | Main model^a^ | Full adjusted model^b^ |
| --- | --- | --- | --- |
|  | OR (95%CI) | OR (95%CI) | OR (95%CI) |
| Sensitivity analysis 1 |  |  |  |
| Noise level ≥ 85 dB(A) (Lex,8 h) | 0.93(0.38-2.30) | 1,22(0.45-3.29) | 1.08(0.39-2.96) |
| CNE ≥ 85 dB(A)-years | 1.42(1.13-1.80) | 1.28(1.00-1.66) | 1.19(0.92-1.55) |
| Sensitivity analysis 2 |  |  |  |
| Noise level ≥ 80 dB(A) (Lex,8 h) | 2.30(1.77-2.99) | 2.30(1.77-2.99) | 2.25(1.72-2.93) |
| CNE ≥ 80 dB(A)-years | 1.50(1.19-1.89) | 1.45(1.12-1.87) | 1.38(1.06-1.79) |
| Noise level ≥ 85 dB(A) (Lex,8 h) | 0.96(0.39-2.37) | 1.25(0.46-3.38) | 1.10(0.40-3.04) |
| CNE ≥ 85 dB(A)-years | 1.45(1.15-1.84) | 1.31(1.02-1.70) | 1.21(0.93-1.58) |

Note: Sensitivity analysis 1 investigated the association of a noise level ≥ 85 dB (A) (Lex,8 h) and CNE ≥ 85 dB(A)-years with hypertension. Sensitivity analysis 2 investigated the association of noise level ≥ 80 dB (A) (Lex,8 h), noise level ≥ 85 dB (A) (Lex,8 h), CNE ≥ 80 dB(A)-years and CNE ≥ 85 dB(A)-years with hypertension after excluding participants who had a history of hypertension or were currently taking antihypertensive medication.

Abbreviation: TG: triglyceride, BMI: body mass index, TC: total cholesterol, HDL: high-density lipoprotein, LDL: low-density lipoprotein, CNE: cumulative noise exposure.

a: Family history of hypertension, smoking, BMI, toxin exposure, dust exposure and duration of noise exposure were adjusted for noise level. Family history of hypertension, smoking, BMI, toxin exposure and dust exposure were adjusted for CNE.

b: Family history of hypertension, smoking, toxin exposure, dust exposure, the duration of noise exposure, TC, TG, HDL and LDL were adjusted for noise level. Family history of hypertension, smoking, toxin exposure, dust exposure, TC, TG, HDL and LDL were adjusted for CNE.

Table 3 results of main model of noise exposure and hypertension stratified by confounding factors (China, 2013).

|  | Noise level ≥ 80 dB(A) (Lex,8 h) | |  | CNE ≥ 80 dB(A)-years | |  |  |
| --- | --- | --- | --- | --- | --- | --- | --- |
| Variable | Case/total | OR (95%CI) | P -interaction | Case/total | OR (95%CI) | P -interaction |  |
| Smoking | |  |  | 0.061 |  |  | 0.040* |
| no | | 92/179 | 3.36(2.24-5.04) |  | 148/397 | 2.08(1.40-3.10) |  |
| yes | | 124/226 | 2.04(1.45-2.86) |  | 207/560 | 1.20(0.86-1.67) |  |
| Family history of hypertension | |  |  | 0.572 |  |  | 0862 |
| no | | 5/10 | / |  | 7/16 | / |  |
| yes | | 211/435 | 2.47(1.90-3.20) |  | 348/941 | 1.51(1.17-1.95) |  |
| Dust exposure | |  |  | 0.116 |  |  | 0.024* |
| no | | 126/229 | 3.14(2.16-4.56) |  | 185/424 | 2.08(1.40-3.09) |  |
| yes | | 90/216 | 2.47(1.69-3.62) |  | 170/533 | 1.28(0.91-1.80) |  |
| Toxin exposure | |  |  | 0.931 |  |  | 0.290 |
| no | | 50/150 | 2.92(1.82-4.69) |  | 93/391 | 1.22(0.77-1.95) |  |
| yes | | 166/295 | 2.68(1.94-3.69) |  | 262/566 | 1.75(1.29-2.38) |  |
| BMI (kg/m2) | |  |  |  |  |  |  |
| 18.5-23.9 | | 31/136 | 2.54(1.51-4.29) |  | 48/328 | 1.06(0.65-1.73) |  |
| <18.5 | | 1/12 | 1.36(0.02-83.12) | 0.607 | 3/27 | 4.15(0.25-68.93) | 0.497 |
| ≥24.0 | | 184/297 | 2.49(1.84-3.36) | 0.438 | 304/602 | 1.69(1.25-2.28) | 0.102 |
| Duration of noise exposure | |  |  | 0.645 |  |  |  |
| ≤ 4 year | | 135/269 | 2.66(1.96-3.61) |  |  |  |  |
| ＞4 year | | 81/176 | 2.24(1.36-3.68) |  |  |  |  |

Abbreviation: BMI: body mass index.
